# Association between Reported Sleep Disorders and Behavioral Issues in Children with Myotonic Dystrophy Type 1—Results from a Retrospective Analysis in Italy

**DOI:** 10.3390/jcm13185459

**Published:** 2024-09-14

**Authors:** Federica Trucco, Andrea Lizio, Elisabetta Roma, Alessandra di Bari, Francesca Salmin, Emilio Albamonte, Jacopo Casiraghi, Susanna Pozzi, Stefano Becchiati, Laura Antonaci, Anna Salvalaggio, Michela Catteruccia, Michele Tosi, Gemma Marinella, Federica R. Danti, Fabio Bruschi, Marco Veneruso, Stefano Parravicini, Chiara Fiorillo, Angela Berardinelli, Antonella Pini, Isabella Moroni, Guja Astrea, Roberta Battini, Adele D’Amico, Federica Ricci, Marika Pane, Eugenio M. Mercuri, Nicholas E. Johnson, Valeria A. Sansone

**Affiliations:** 1 The NeMO Clinical Center in Milan, 20162 Milan, Italy; federica.trucco@unige.it (F.T.); andrea.lizio@centrocliniconemo.it (A.L.); elisabetta.roma@centrocliniconemo.it (E.R.); alessandra.dibari@centrocliniconemo.it (A.d.B.); francesca.salmin@gmail.com (F.S.); emilio.albamonte@centrocliniconemo.it (E.A.); jacopo.casiraghi@centrocliniconemo.it (J.C.); susanna.pozzi@centrocliniconemo.it (S.P.); stefano.becchiati@centrocliniconemo.it (S.B.); 2 Department of Neurorehabilitation, University of Milan, 20122 Milan, Italy; 3 Paediatric Neurology and Muscular Diseases Unit, Istituto di Ricovero e Cura a Carattere Scientifico Istituto Giannina Gaslini, Department of Neurosciences, Rehabilitation, Ophthalmology, Genetics, Maternal and Child Health, University of Genova, 16132 Genoa, Italy; 4 Fondazione Policlinico Universitario Agostino Gemelli, IRCCS Università Cattolica del Sacro Cuore, 00136 Roma, Italy; laura.antonaci@guest.policlinicogemelli.it (L.A.); marika.pane@policlinicogemelli.it (M.P.); eugeniomaria.mercuri@unicatt.it (E.M.M.); 5 Department of Sciences of Public Health and Pediatrics, University of Turin, 10124 Turin, Italy; anna.salvalaggio@unito.it (A.S.); federica.ricci@unito.it (F.R.); 6 UOS Malattie Muscolari e Neurodegenerative—Ospedale Pediatrico Bambino Gesù, 00165 Roma, Italy; michela.catteruccia@opbg.net (M.C.); michele.tosi@opbg.net (M.T.); adele2.damico@opbg.net (A.D.); 7 Department of Developmental Neuroscience, IRCCS Fondazione Stella Maris, 56128 Calambrone Pisa, Italy; gemma.marinella@fsm.unipi.it (G.M.); guja.astrea@fsm.unipi.it (G.A.); roberta.battini@fsm.unipi.it (R.B.); 8 Department of Pediatric Neurosciences, Fondazione IRCCS Istituto Neurologico Carlo Besta, 20133 Milan, Italy; rachele.danti@istituto-besta.it (F.R.D.); isabella.moroni@istituto-besta.it (I.M.); 9 Unit of Child Neuropsychiatry, IRCCS Istituto Giannina Gaslini and DINOGMI, University of Genova, 16132 Genova, Italy; marc.veneruso@gmail.com (M.V.); chiara.fiorillo@edu.unige.it (C.F.); 10 Child and Adolescent Neuromuscular Disorders Unit, IRCCS Mondino Foundation, 27100 Pavia, Italy; stefano.parravicini01@universitadipavia.it (S.P.); angela.berardinelli@mondino.it (A.B.); 11 Department of Brain and Behavioral Sciences, University of Pavia, 27100 Pavia, Italy; 12 Pediatric Neuromuscular Unit, UOC Neuropsichiatria dell’età Pediatrica, IRCCS Istituto delle Scienze Neurologiche di Bologna, 40139 Bologna, Italy; antonella.pini@isnb.it; 13 Department of Clinical and Experimental Medicine, University of Pisa, 56126 Pisa, Italy; 14 Department of Neurology, Virginia Commonwealth University, Richmond, VA 23298, USA; nicholas.johnson@vcuhealth.org

**Keywords:** congenital myotonic dystrophy type 1, childhood myotonic dystrophy type 1, sleep-related breathing disorders, obstructive sleep apnea, excessive daytime sleepiness

## Abstract

**Background:** Sleep disorders have been poorly described in congenital (CDM) and childhood (ChDM) myotonic dystrophy despite being highly burdensome. The aims of this study were to explore sleep disorders in a cohort of Italian CDM and ChDM and to assess their association with motor and respiratory function and disease-specific cognitive and behavioral assessments. **Methods:** This was an observational multicenter study. Reported sleep quality was assessed using the Pediatric Daytime Sleepiness Scale (PDSS) and Pediatric Sleep Questionnaire (PSQ). Sleep quality was correlated to motor function (6 min walk test, 6MWT and grip strength; pulmonary function (predicted Forced Vital Capacity%, FVC% pred.); executive function assessed by BRIEF-2; autism traits assessed by Autism Spectrum Screening Questionnaire (ASSQ) and Repetitive Behavior Scale-revised (RBS-R); Quality of life (PedsQL) and disease burden (Congenital Childhood Myotonic Dystrophy Health Index, CCMDHI). **Results:** Forty-six patients were included, 33 CDM and 13 ChDM, at a median age of 10.4 and 15.1 years. Daytime sleepiness and disrupted sleep were reported by 30% children, in both subgroups of CDM and ChDM. Daytime sleepiness correlated with autism traits in CDM (*p* < 0.05). Disrupted sleep correlated with poorer executive function (*p* = 0.04) and higher disease burden (*p* = 0.03). **Conclusions:** Sleep issues are a feature of both CDM and ChDM. They correlate with behavioral issues and impact on disease burden.

## 1. Introduction

Myotonic dystrophy type 1 (DM1) is an autosomal dominant multisystem disorder affecting skeletal, cardiac, and smooth muscle, as well as the eye, endocrine, and central nervous system (CNS) with a variable age of onset [1,2,3]. The disease is clinically classified as congenital, childhood, adult- and late-onset [4,5] based on the age at which symptoms appear and depending on the distinct features displayed by each phenotype [6,7,8].

The central nervous system (CNS) is involved in both pediatric and adult-onset DM1. Impaired executive function, issues with verbal and visuospatial memory as well as deficits in processing speed have been described in adults [9] and children [10]. However, individuals with pediatric-onset DM1 have specific neurodevelopmental issues, which are not seen in adult-onset DM1. Autism spectrum disorder (ASD) and attention-deficit/hyperactivity disorder (ADHD) are more prevalent in ChDM, with up to 30% of patients being diagnosed [11,12], while intellectual impairment is more frequent in the congenital form [13]. Up to 90% of children with CDM [14] and 30% to 40% of children with ChDM [7,10] have impaired cognition.

Sleep issues are also a prominent feature of DM1. They result from the interplay between structural and functional abnormalities in the CNS, skeletal muscle, and upper airway. In adults with DM1, sleep issues have been extensively studied. Over 80% of adult patients with DM1 reported suffering from sleep disorders [15,16]. Excessive daytime sleepiness (EDS) [15,17,18], disruption of sleep–wake cycle patterns, such as narcolepsy-like features [19,20,21,22], and-sleep related breathing abnormalities [23,24] are known to be the most common and burdensome symptoms in adults living with DM1 [16].

Conversely, data on sleep disorders in the pediatric population are limited. There are indications that sleep issues represent a significant co-morbidity [7,8,25,26,27] for patients and families. Sleep issues were identified in 66% of 21 children with DM1 who were surveyed [26]. EDS and sleep fragmentation were described in over half of this cohort. EDS was found to significantly impact the perceived quality of life in children with DM1 [27]. In addition to primary sleep disorders, sleep-disordered breathing requiring non-invasive ventilation (NIV) is also prevalent in pediatric-onset DM1 [25,28].

So far, however, the association between sleep issues and neurodevelopmental disorders in CDM and ChDM has not been extensively investigated.

The main aim of this study was to describe sleep disorders through qualitative and quantitative assessments in a multicenter large cohort of Italian children with congenital and childhood-onset DM1 and to determine whether any association existed between sleep issues and neuromuscular disability, neurodevelopmental traits like autism and frontal executive functions, disease burden, and perceived quality of life.

## 2. Materials and Methods

### 2.1. Study Design

This was a retrospective cross-sectional observational multicenter study including consecutive patients with a diagnosis of either CDM or ChDM followed at the NeMO Clinical Center in Milan and at 8 additional Italian neuromuscular centers (Istituto Neurologico Carlo Besta, Milano; Istituto Neurologico Casimiro Mondino, Pavia; Istituto Giannina Gaslini, Genova; Istituto delle Scienze Neurologiche, Bologna; Fodazione Stella Maris, Pisa; Fondazione Policlinico Universitario Agostino Gemelli, Roma; Ospedale Pediatrico Bambin Gesù, Roma; Ospedale Regina Margherita, Torino) between Jun 2019 and Dec 2022.

This study was supported by a grant to VS (Telethon-UILDM grant GUP19002) and was approved by the Institutional Review Board (Ethics Committee) at each participating study site. Written informed consent was obtained from all participants (or guardians of participants) in the study (consent for research).

### 2.2. Study Population

Pediatric patients (age < 18 years) were enrolled with a genetic test confirming the diagnosis of DM1, defined as the detection of CTG repeat expansion in the *DMPK* gene, as per standard guidelines [29].

Patients’ age, gender, diagnosis of either CDM or ChDM based on the onset of symptoms, and class of CTG expansion were collected and correlated with reported sleep issues.

The diagnosis of CDM was defined as the onset of symptoms within the newborn period (first 30 days of life). Patients with onset of symptoms after day 30 from birth and before the age of 10 years were classified as ChDM [30]. The CTG expansion was classified according to the number of CTG repeats, as E1 = 200–500 CTG, E2 = 500–1000 CTG, E3 = 1000–1500 CTG, E4 > 1500 CTG [31].

All patients underwent qualitative sleep assessments and age-appropriate cognitive and behavioral tests administered and scored by trained clinical psychologists, quantitative sleep assessments (i.e., overnight sleep studies) administered and scored by certified sleep physician, as well as neuromuscular and respiratory assessment conducted by trained and certified physiotherapists.

All patients were consecutively enrolled by the centers as part of a natural history study. Only patients who had at least one reliable sleep assessment completed were included in this study.

### 2.3. Sleep Assessments

#### 2.3.1. Reported Sleep Issues—Qualitative Assessments

Questionnaires were administered to children if appropriate for age and/or for the child’s cognitive abilities. In all other cases, the questionnaires were filled in by parents. Supervision in the completion of the questionnaire was offered to parents if they were also affected.

##### Pediatric Daytime Sleepiness Scale (PDSS)

The PDSS is a self-reported 8-item scale that assesses daytime sleepiness in children as young as 11 years of age and adolescents. It has been previously used to assess the consequence of sleep disruption due to sleep-disordered breathing and in studies with patients affected by severe epilepsy [32,33]. A value above 15 has been proposed as a cut-off associated with poor sleep quality [34].

The reliability of the PDSS in terms of internal consistency (Cronbach’s alpha) for the total 8-item scale was 0.80. Considering the Brazilian Portuguese validation, the internal consistency analysis showed a Cronbach’s alpha of 0.78, and the questionnaire reported an adequate reproducibility. Test–retest reliability and internal consistency were acceptable in the Chinese version [32,35].

##### Pediatric Sleep Questionnaire (PSQ)

The PSQ is a parental questionnaire that includes subscales for snoring, sleep-disordered breathing, restless leg syndrome, parasomnias, narcolepsy, insomnia, daytime inattention and hyperactivity, as well as daytime sleepiness. Good internal consistency and test–retest reliability have been demonstrated with sleep-related breathing disorders in the general pediatric populations [36]. The score ranges from 0 to 1. PSQ has been previously used in children with CDM [25] and with other neurodevelopmental and respiratory disorders [37,38,39].

Previous data suggest that values above 0.33 are most likely associated with sleep disruption impacting daytime activities [36,40,41].

The reliability of the PSQ in terms of internal consistency (Cronbach’s alpha) was good. Among group A subjects (development cohort), Cronbach’s alpha for each scale was: snoring scale, 0.86; sleepiness scale, 0.66; behavior scale, 0.84; and SRBD scale, 0.89. Among group B subjects (validity cohort), Cronbach’s alpha for each scale was: snoring scale, 0.86; sleepiness scale, 0.77; inattention/hyperactivity scale, 0.83; and SRBD scale, 0.88. Regarding the test–retest analysis, a Spearman correlation coefficient r of 0.92 for the snoring scale (*p* < 0.0001), 0.66 for the sleepiness scale (*p* = 0.0010), 0.83 for the behavior scale (*p* < 0.0001), and 0.75 for the SRBD scale (*p* < 0.0001) revealed a good reproducibility of the questionnaire [36].

#### 2.3.2. Sleep Disordered Breathing

To support the qualitative perception of disturbed sleep, when available, results from objective sleep assessments were collected. Only sleep studies which were conducted at the same time or during the same admission of the qualitative assessments were included in this study.

Patients underwent overnight monitoring of SpO_2_ during sleep via nocturnal oximeter (Covidien Nellcor Bedside SpO2, Nellcor, NY, USA).

When available, data gathered from cardiorespiratory polygraphy (Embletta, Natus, Middleton, WI, USA) were also collected in order to quantify the severity of sleep-related apneas and hypopneas. Cardiorespiratory polygraphy was scored according to AASM criteria by a trained sleep specialist [42].

### 2.4. Neuromuscular Assessments

Patients’ motor function was assessed via a combined set of tests validated for neuromuscular disorders [43,44].

*The 6 min walk test (6MWT)*. Patients completed the 6MWT along a 25 m course as per ATS guidelines [45] in accordance with previously validated protocols for pediatric neuromuscular disorders [46,47].

*Grip and pinch strength.* The maximum voluntary isometric contraction (MVIC) grip strength and pinch were measured using a JAMAR+ digital hand dynamometer (Sammons Preston, Warrenville, Illinois, USA) and digital pinch dynamometer on each right and left hand with the arm resting at their side in a seated position at 90 degrees’ elbow flexion and neutral forearm supination. The best out of three trials was recorded [47].

### 2.5. Pulmonary Function and Requirement for Ventilatory Support

Pulmonary function was assessed via spirometry at each site by trained respiratory physiotherapists. Spirometry was performed in upright position in compliance with ATS/ERS guidelines. The best of 3 efforts deemed reliable by the operator was recorded [48].

Percent-predicted Forced Vital Capacity (pred FVC%), peak cough flow (PCF) absolute (liters per minute, L/min) was collected via spirometry performed with the patient tested in a sitting position.

Patients’ use of non-invasive ventilation (NIV) and cough device was also collected.

### 2.6. Cognitive and Behavioral Assessments

Behavior Rating Inventory of Executive Function (BRIEF) is used for those from age 2 years and 6 months and BRIEF-P for children younger than 2 years and 6 months. The BRIEF is a parent-proxy rating form of executive function skills in every-day settings such as school, home, and social situations (e.g., task planning and organization, multi-tasking, self-monitoring of behavior). It comprises nine subscales with three index scores: the behavioral regulation index, emotional regulation index, and cognitive regulation index. The BRIEF-2 also provides a Global Executive Composite (GEC) score, which was used for analyses in this work. The total score ranges from 0 to 100, and a score above 60 is considered pathological.

*Autism Spectrum Screening Questionnaire (ASSQ).* The ASSQ is a screening measure designed to assess children aged 7 to 16 years applicable to those with mild cognitive impairment. The ASSQ contains 27 fixed-choice questions that can be answered by parents and teachers to assess symptoms characteristic of autism spectrum disorder (ASD) in children and adolescents. Cut-off scores of 19 and 22 for parent and teacher rating, respectively, are recommended to identify possible ASD [49].

*Repetitive Behavior Scale—Revised (RBS-R).* The RBS-R is a 44-item parent-proxy questionnaire that measures the extent of repetitive behavior in children between 6 and 17 years. RBS-R includes six subscales, each rated on a 4-point scale. Participants provide a global rating of how problematic these repetitive behaviors are overall on a scale from 0 to 3, with 0 not being a problem and 3 representing the worst possible scenario [50].

### 2.7. Reported Quality of Life and Disease Burden

*Pediatric Quality of Life questionnaire (Peds QL).* The PedsQL evaluates a child’s health-related quality of life (HRQoL) with respect to social, emotional, physical, and school functioning. The PedsQL can be administered directly to children for self-reported HRQoL and/or to parents to obtain proxy ratings of a child’s HRQoL, as validated in other neuromuscular conditions [51]. Only the score related to the parent’s reported HRQoL was considered for analysis in this study.

*Congenital and Childhood Myotonic Health Index (CCMDHI).* CCMDHI is a reported measure of disease burden and consists of a parental-proxy questionnaire and an age-appropriate short form (ages 5–7, 8–11, and 12–17). The questionnaires were translated to Italian by a professional translator as per standard practice. The CCMDHI has been developed from the adult version, the Myotonic Dystrophy Health Index [52], considering the specific aspects of the congenital and childhood DM1. The weighted sum of responses is transformed into a percentage of the maximum possible value, with a score of 100 representing the most severe disease burden and a score of zero representing no disease impact.

### 2.8. Statistical Analysis

The distribution of each variable was assessed using Shapiro–Wilk to test the normality and the Levene test to assess the homogeneity of the variance.

Descriptive analysis was carried out using median and interquartile range (IQR) for continuous variables, and number and percentage for dichotomous and categorical ones.

The associations between the scores of qualitative sleep assessments and age, motor, respiratory, and cognitive–behavioral assessments as well as quality of life and disease burden were assessed using the Spearman rank correlation coefficient and the Mann–Whitney U test, as appropriate.

All tests were two-tailed, and a *p*-value < 0.05 was considered as statistically significant. Analyses were performed using SAS 9.3 (SAS Institute Inc., Cary, NC, USA).

## 3. Results

### 3.1. Characteristics of the Study Population

Forty-six subjects were included. Thirty-three children had congenital onset, and thirteen were classified as having childhood-onset DM1. Table 1 describes the demographic and clinical characteristics of the 46 children included in this study.

### 3.2. Reported Sleep Issues

#### 3.2.1. Excessive Daytime Sleepiness

Excessive daytime sleepiness was assessed using the Pediatric Daytime Sleepiness Scale (PDSS) in 40 of the 46 patients, 28 of whom had CDM and 12 had ChDM. The median (IQR) score in the whole population was 12 [9.5–17.5]. Thus, 12 of the 40 patients (30%) scored higher than 15 at PDSS, which is consistent with EDS [34]. Six were children with CDM, and six were children with ChDM, resulting in a prevalence of excessive daytime sleepiness of 21% (6 of 28) in CDM and 50% (6 of 12) in ChDM. There was no significant difference in the rate of EDS between the two groups (*p* = 0.7).

All 12 patients qualifying as having EDS were older than 10 years of age, meaning that none of the children younger than 10 years in the study population scored over 15 at PDSS.

#### 3.2.2. Sleep Disruption

The quality of overnight sleep was assessed using the Pediatric Sleep Questionnaire (PSQ) completed by parents. Results were available in 42 of the 46 patients; 31 were children with CDM and 11 were children with ChDM. The median (IQR) PSQ score was 0.26 [0.18–0.36]. Twelve of the forty-two patients (29%) had a value of PSQ above 0.33, which is considered consistent with disturbed overnight sleep.

The median PSQ value in children with CDM was 0.26 [0.18–0.36] with 26% patients (8 out of 31) scoring higher than the threshold of 0.33. The median PSQ value in children with ChDM was 0.23 [0.14–0.42]. Four of the eleven patients with ChDM (36%) scored above 0.33. There were no significant differences between either the severity or the prevalence of the reported sleep issues between CDM and ChDM.

Considering the clinical thresholds of PDSS (a score above 15 being consistent with daytime sleepiness) and PSQ (a score above 0.33 being consistent with disrupted sleep), 81% of patients had concordant results in the two questionnaires. In detail, 61% of patients did not report daytime sleepiness or disrupted sleep, whilst 20% reported both. Conversely, opposing results from the two questionnaires were found in 19% of patients. Of them, 8% of patients reported daytime sleepiness without disrupted sleep, whilst 11% reported disrupted sleep without daytime sleepiness (see Table 2).

### 3.3. Sleep-Related Breathing Disorders

To support the qualitative perception of sleep issues, objective sleep assessments were collected. Sleep studies were included for analysis only if they were conducted during the same admission when qualitative sleep assessments were collected. Sleep studies fulfilling these criteria were available for 17 of the 46 patients; 10 were children with CDM and 7 were children with ChDM.

Four patients had a cardiorespiratory sleep study associated with oximetry. One patient had a normal study, being the total apnea–hypopnea index (AHI) 0.2 per hour. The remaining three patients only had mild sleep-disordered breathing, with a total AHI ranging from 2.1 to 3.2 per hour. Most events were scored as central and were not associated with significant desaturations per hour.

Of the 13 patients who had an overnight oximetry alone, 9 had a normal oximetry with a mean overnight SpO2 value of 97%. In five patients, the only finding was the number of desaturations per hour (oxygen desaturation index, ODI), above three per hour, which is considered mildly elevated compared to normative data in healthy children [53].

### 3.4. Motor, Respiratory, and Cognitive–Behavioral Function

All patients were ambulant, with a median distance of 459 (400–523) meters at 6MWT. Children with ChDM were able to walk longer than children with CDM, with a median of 506 and 423 m, respectively.

Grip and pinch strength of children with CDM were half the maximal strength of children with ChDM on both the dominant and non-dominant sides. The absolute values of grip and pinch strength shown in Table 3 were consistent with those previously reported in the literature [25].

Regarding respiratory function, spirometry was feasible in 34 of the 46 patients, due to difficulties related to complying with the task. Of the 34 patients able to perform spirometry, 19 were CDM and 15 were ChDM. The median value of predicted FVC% was normal in ChDM (82%, range 72–95%) and only mildly reduced in CDM (68%, range 36–79%).

The assessment of cough strength was available in 20 patients. A peak cough flow below 160 L/min, (weak cough) was found in 4/11 (36%) CDM and in none (0%) of the 9 ChDM patients.

NIV was prescribed in four patients due to reduced levels of mean SpO2 (94–96%) associated with a restrictive lung pattern at spirometry (FVC% pred. 40% to 45%).

The behavioral evaluations were completed by 30 patients. Thirty-three percent of them had impaired executive functions assessed via the BRIEF-2 Global Executive Composite (GEC). In detail, 44% of children with CDM and 17% of children with ChDM had a score of BRIEF-2 above 60.

None of the patients had values of either the Autism Spectrum Screening Questionnaire (ASSQ) or the Repetitive Behavior Scale revised (RBS-R) suggesting behavioral traits of autism spectrum disorders requiring further assessments.

Table 3 summarizes the results of the assessments of motor, respiratory, and cognitive–behavioral function in the study population.

### 3.5. Correlations between Reported Sleep Issues and Disease Features

Excessive daytime sleepiness assessed via PDSS was significantly related to gender in the overall population. Female patients scored higher than males (median PDSS score 15.00 [10.00–22.00] and 11.00 [9.00–12.00] in females and in males, respectively; *p* = 0.0301), suggesting a more severe daytime sleepiness in the former. In patients with CDM, the severity of reported daytime sleepiness positively correlated with the presence of autistic traits assessed via the ASSQ scale (*p* < 0.05), while no correlation was found between reported daytime sleepiness and patients’ age, executive function assessed via BRIEF-2, or parents’ reported quality of life. In patients with ChDM, the reported excessive daytime sleepiness did not correlate with the autism scale, age, motor function, executive function, or quality of life and disease burden. No differences were found in the median value of PDSS between patients with or without sleep-disordered breathing, defined as an oxygen desaturation index above or below 3/h.

Reported sleep disruption, defined by a PSQ score above 0.33, was significantly correlated to an impaired executive function expressed by a BRIEF-2 score above 60 (*p* = 0.04) and to a higher perceived disease burden expressed by higher scores of CCMDHI (*p* = 0.03). Reported sleep disruption did not correlate with either age, gender, motor, or pulmonary function (FVC% pred.) in the overall population or in children with CDM or ChDM.

## 4. Discussion

The characterization of sleep issues in pediatric DM1 is still relatively limited [25,26,27,28]. Sleep issues, both sleep-related breathing disorders and primary sleep abnormalities, have been investigated in relatively small cohorts of children with DM1, without taking into account any difference between the congenital and childhood-onset phenotypes. Also, the association between sleep disorders and neurodevelopmental traits, typical of the disease, has not been explored so far.

In this study, over 30% of children reported excessive daytime sleepiness. The severity of excessive daytime sleepiness impacting daily activities was assessed via the Pediatric Daytime Sleepiness Scale (PDSS). The prevalence of daytime sleepiness was in line with previous works conducted using PDSS on a cohort of children with CDM [25]. The prevalence of daytime sleepiness, measured by the modified pediatric Epworth sleepiness scale, was 50% in another cohort of 21 children with DM1 [26]. Conversely, as opposed to what was found by Ho et al. [27], in this study, no correlation was found between excessive daytime sleepiness measured via PDSS and measures of quality of life (PedsQL) or disease burden (CCMDHI).

Excessive daytime sleepiness was found to be associated with gender, with females being more affected than males. Females in this cohort had a more severe reported daytime sleepiness than males, possibly due to a different behavioral response to sleepiness resulting in apathy more than in hyperactivity. Also, all patients reporting a PDSS score consistent with excessive daytime sleepiness were older than 10 years of age. This second finding may either suggest that sleepiness progresses along the course of the disease or that as children grow and their functionality improves, as per natural history of CDM for instance, sleepiness has a greater impact on the activity of daily living. Also, pathological sleepiness in younger children may present with peculiar features such as hyperactivity or changes in sleep–wake rhythm, rather than with observed difficulties in falling asleep. The presence of an autistic trait assessed via ASSQ was associated with a more severe reported excessive daytime sleepiness. Conversely, the presence of sleep-related breathing disorders did not impact the severity of excessive daytime sleepiness.

Another aspect this study sought to investigate was the prevalence of sleep disruption, measured by the Pediatric Sleep Questionnaire (PSQ). Twenty-nine percent of children in the study population reported disrupted sleep. The prevalence was moderately higher in children with ChDM (36%) than with CDM (26%), yet not statistically different (*p* = 0.7). Scores of PSQ above 0.33 did correlate with a higher reported disease burden expressed by CCMDHI.

The severity of reported sleep issues did not correlate, in this study, with patients’ CTG expansion in either CDM or ChDM.

The presence and features of sleep-disordered breathing were only available in a sub-cohort of 17 patients. This was due to the poor tolerance to the overnight equipment by some of the most severely affected children. The ability of children with significant cognitive difficulties to comply with recommended standards of care should be evaluated when assessing adherence to these standards. Despite these limitations, sleep-disordered breathing identified as respiratory polygraphy or defined as an elevated oxygen desaturation index (ODI) at overnight oximetry was identified in 9 out of 17 (53%) patients, in line with previous studies reporting a prevalence of sleep apnea, mainly obstructive, ranging from 29% [26] to 42% [28].

The possible contribution of sleep-disordered breathing to the severity of daytime sleepiness in DM1 as the result of sleep fragmentation is controversial. In the work by Quera Salva et al., respiratory events resulted in fatigue (76%) and somnolence (52%) due to sleep fragmentation [26]. On the other hand, daytime sleepiness has been reported independently from the evidence of sleep-related breathing disorders [27]. In this study cohort, no differences were found in the severity of reported excessive daytime sleepiness between patients with or without sleep-disordered breathing. Notably, given the behavioral and cognitive profile of these patients, discrepancies between subjective and objective measures of sleepiness are to be considered. For instance, in 63 patients with adult-onset DM1, 48% had a short sleep onset in the multiple sleep latency test but only 33% reported sleepiness on the Epworth Sleepiness scale (ESS score > 10) [54].

It is also worth noting that, unlike other neuromuscular disorders, the severity of sleep-disordered breathing in children with DM1 seems not to be strictly associated with the severity of the restrictive pulmonary function. Pulmonary function does not seem to deteriorate over time in children with DM1 [28].

Finally, 4 of the 17 patients required NIV initiation. All of them were children with CDM. They had reduced mean overnight levels of SpO2 (ranging from 94 to 96%) associated with a restrictive lung disease (FVC% pred. 40% to 45%). This is in line with the experience of a French network reporting, as a main indication of NIV (in 31% of patients) in DM1, the presence of overnight abnormal gas exchange with either hypoxia or hypercapnia [55]. The percentage of children with DM1 who had an indication to NIV requirement widely varies across different cohorts of pediatric DM1, ranging from 38% [28] to 3% [56].

NIV in DM1 has proven effective in treating overnight gas exchange [23,57] and, ultimately, in prolonging patients’ long-term survival [58,59,60]. However, the compliance to NIV remains challenging in DM1 patients, irrespective of their age and severity [28,55]. Interestingly, in DM1 patients, even if not using NIV, the alteration in nocturnal gas exchange does not progress significantly.

The challenges in testing young subjects with severe cognitive impairment are still limiting the widespread systematic assessment of respiratory function. Also, the high prevalence of behavioral difficulties further impacts sleep quality, making the investigation of sleep issues in pediatric DM1 exceptionally daunting. Care recommendations in this patient population and the increasing awareness in DM1 [30] have, at least in part, contributed to the fact that patients with CDM and ChDM now survive more easily into adulthood. However, the natural history of these subtypes is far from being defined, as the progression of the disease differs from the classic adult-onset DM1.

This work results from a collaborative network of tertiary care neuromuscular centers looking after families with DM1. This study was specifically focused on sleep disorders and included a broad cohort of well-characterized children with CDM and ChDM.

However, limitations should also be acknowledged. These include the limited number of sleep studies available. Given the retrospective nature of this study, the results of the sleep study were not retrieved for all patients or were not completed/recorded by each center at the time of admission. Another limitation may be represented by the lack of a control population.

In conclusion, this study, conducted on 46 well-characterized children with DM1, confirms that sleep issues, investigated via qualitative and quantitative measures, are a feature of the disease and that they are associated with autistic traits, dysexecutive function, and to a higher disease burden. The prevalence and severity of both reported EDS and disrupted sleep did not differ between CDM and ChDM.

Prospective longitudinal studies will contribute to better defining the progression of sleep issues and how they impact functional neurodevelopmental and neuromotor outcomes in the long term. Other studies aiming to identify clinical and biological biomarkers targeting sleep pathways are planned in patients with pediatric-onset DM1. In preparation for clinical trials, sleep can indeed represent an important and measurable aspect of the disease, particularly in the era of novel disease-modifying treatment targeting both muscles and the central nervous system.

## Figures and Tables

**Table 1:** Study population.

	Overall (*n* = 46)	CDM (*n* = 33)	ChDM (*n* = 13)
Age, median (IQR), years	11.96 [8.23–15.16]	10.40 [8.00–12.72]	15.09 [8.83–16.61]
Gender, male (%)	22 (47.82)	17 (51.51)	5 (38.46)
Class of CTG expansion			
E1	15 (32.61)	7 (21.21)	8 (61.54)
E2	15 (32.61)	12 (36.36)	3 (23.08)
E3	13 (28.26)	12 (36.36)	1 (7.69)
E4	3 (6.52)	2 (6.06)	1 (7.69)

The class of CTG expansion are classified E1 = 200–500 CTG, E2 = 500–1000 CTG, E3 = 1000–1500 CTG, E4 > 1500 CTG. Savic Pavicevic D. et al. Biomed Res Int 2013 [31]. Abbreviations. IQR—interquartile range; CDM—congenital onset myotonic dystrophy; ChDM—childhood onset myotonic dystrophy.

**Table 2:** Reported sleep issues in the study population.

	Overall	CDM	ChDM	*p* Value
PDSS, median (IQR)	12.00 [9.50–17.50]	12.00 [10.00–14.50]	15.00 [8.50–22.50]	0.5535
PDSS Classification, *n* (%)				0.6992
≤15 (No report of daytime sleepiness)	28 (70.00)	22 (78.57)	6 (50.00)	
>15 (Daytime sleepiness)	12 (30.00)	6 (21.43)	6 (50.00)	
PSQ, median (IQR)	0.26 [0.18–0.36]	0.26 [0.18–0.36]	0.23 [0.14–0.42]	0.7305
PSQ Classification, *n* (%)				0.6992
≤0.33 (No report of disrupted sleep)	30 (71.43)	23 (74.19)	7 (63.64)	
>0.33 (Disrupted sleep)	12 (28.57)	8 (25.81)	4 (36.36)	

Abbreviations. PDSS: Pediatric Daytime Sleepiness Scale; PSQ: Pediatric Sleep Questionnaire. A PDSS score above 15 was consistent with daytime sleepiness; a PSQ score above 0.33 was consistent with disrupted sleep. A *p* value below 0.05 was considered statistically significant.

**Table 3:** Motor, respiratory, cognitive–behavioral features and quality of life of the study population.

	Overall	CDM	ChDM
Motor Function			
6MWT, median (IQR) meters	458.90 [400.00–523.00]	423.00 [400.00–487.00]	505.50 [422.00–533.00]
Grip_dx, median (IQR) kg	9.15 [5.30–16.15]	5.65 [2.14–10.50]	13.80 [7.80–23.00]
Grip_sx, median (IQR) kg	9.90 [5.00–17.10]	5.20 [1.90–11.30]	14.05 [6.00–17.10]
Pinch_dx, median (IQR) kg	2.50 [1.80–4.30]	1.80 [1.60–2.14]	3.85 [3.00–4.30]
Pinch_sx, median (IQR) kg	2.50 [2.00–3.50]	2.00 [1.10–2.70]	3.50 [2.50–4.00]
Respiratory Function			
FVC% pred. upright, median (IQR) value	74.00 [68.00–88.00]	68.00 [36.00–79.00]	81.50 [72.00–94.50]
FVC% pred. Classification, *n* (%)			
≥80% (normal)	8 (38.10)	2 (22.22)	6 (50)
<80% (restrictive lung disease)	13 (61.90)	7 (77.78)	6 (50)
PCF, median (IQR) value (L/min)	202.40 [59.40–329.20]	144.00 [64.50–340.80]	246.10 [177.10–300.00]
Executive function			
BRIEF2 GEC, median (IQR) score	55.50 [50.00–63.00]	60.00 [48.00–64.00]	54.50 [50.50–58.50]
BRIEF2 GEC Classification, *n* (%)			
≤60 (normal)	20 (66.67)	10 (55.56)	10 (83.33)
>60 (impaired)	10 (33.33)	8 (44.44)	2 (16.67)
Autism			
ASSQ, median (IQR) score	11.00 [5.00–16.50]	13.00 [5.50–18.00]	7.00 [3.00–12.00]
RBS-R, median (IQR) score	6.00 [1.00–14.00]	9.00 [2.00–18.00]	2.00 [0.00–8.00]
Quality of Life and disease burden			
HRQoL Parent, median (IQR)	68.75 [40.62–87.50]	56.25 [38.00–96.80]	70.28 [59.38–75.00]
PedsQL Parent, median (IQR)	70.58 [59.00–82.00]	71.41 [59.00–83.33]	66.67 [59.00–73.33]
CCMDHI Total score, median (IQR)	10.10 [2.17–27.05]	10.10 [2.31–32.81]	8.84 [1.75–15.25]

Abbreviations. 6MWT: 6 min walk test; FVC%: Forced Vital Capacity percent of predicted; PCF: Peak Cough Flow; ASSQ: Autism Spectrum Screening Questionnaire; RBS-R: Repetitive Behavior Scale, revised; BRIEF-2: Behavior rating Inventory of executive Function, second edition; HRQoL: Health-related quality of life; PedsQL: quality of life; CCMDHI: Congenital and Childhood Myotonic Dystrophy Health Index.

## Data Availability

The data presented in this study are available on request from the corresponding author in an anonymized form only.
